# P054 More than meets the eye: a management dilemma in severe refractory disease and reproductive care

**DOI:** 10.1093/rap/rkag113.084

**Published:** 2026-11-03

**Authors:** Alex Connew

**Affiliations:** Southampton General Hospital, Southampton, United Kingdom

## Abstract

**Introduction:**

A case of Takayasu’s arteritis presenting atypically with visual symptoms. Treatment has required complex decision making because of both the high burden of disease, including active inflammation and vascular complications, and the need to consider the patient’s reproductive goals, as she has not yet completed her family. This case demonstrates MDT working and a patient-centred holistic approach to management.

**Case description:**

A 34-year-old female of South Asian descent, with a background of hypothyroidism, initially noted visual symptoms. This manifested as episodic “brightness” and “floaters”. She attended accident and emergency. Physical examination revealed blood pressure of 77/40mmHg, sinus tachycardia of 120 beats per minute and a systolic murmur. Her CRP was 20, TFTs normal, with no other blood abnormalities, with a normal echocardiogram. Subsequently, she was discharged. Her visual symptoms progressed and were described as episodic “dimming”. An Ophthalmology assessment showed retinal vasculitis and prompted a referral to rheumatology. Absent radial pulses and upper limb claudication symptoms were noted. A CT Angiogram of carotids and circle of Willis showed diffuse wall thickening and significant narrowing in the brachiocephalic trunk, both common carotid and subclavian arteries. Total occlusion of the middle and distal segment of the right common carotid artery. A diagnosis of Takayasu’s arteritis was made.

Her initial treatment consisted of pulsed IV methylprednisolone, followed by a weaning regime of oral prednisolone. Azathioprine, tocilizumab and aspirin were also started. The patient has not completed her family, and this was taken into consideration for initial treatment. Azathioprine was later stopped due to hair loss. This was switched to methotrexate. Which was held periodically and reduced due to intermittent transaminitis.

6 months after diagnosis she developed an MCA infarct requiring thrombolysis.

Cyclophosphamide was considered, as well as egg harvesting (prior to treatment) however this was declined by the patient. The pre-conception team have since advised against pregnancy due to high risk of maternal death.

Currently the patient is having ongoing claudication symptoms. Raising the questions of ongoing activity versus damage. MDT discussions have considered applying for an Individual Funding Request for a TNF inhibitor/ increasing tocilizumab. Vascular discussions have given the view that intervention would be very high risk and decided against currently.

**Discussion:**

As a large vessel vasculitis, it is unusual for a patient to present with visual symptoms and retinal vasculitis. Raising awareness will allow more clinicians to consider Takayasu’s arteritis as a differential in those presenting similarly.

This case highlights the balance between aggressive immunosuppression in those with a high burden of disease whilst also considering reproductive goals. This case explores options available to those with a high burden of disease, in terms of medications and measures to protect reproductive opportunities, such as egg harvesting. Also addressed is the difficult decision to advise against pregnancy and to prioritise the women’s health. Emphasising a fundamental principle of pre-conception medicine; a healthy mother, is more likely to lead to a healthy pregnancy/baby.

This case also explores not just medical but also surgical treatments. As well as MDT working between the pre-conception service, vascular surgical team and the rheumatology team.

In addition to highlighting the challenges with difficult to treat Takayasu’s arteritis, emphasised are the limited options available to our Takayasu’s patients, compared to other illnesses that we look after. Individual Funding Requests can be a lengthy process and can lead to delays in treatment.

**Key learning points:**

• Visual symptoms and retinal vasculitis should raise suspicion of Takayasu’s arteritis. Prompting further questioning +/- investigations.

• This case highlights the complexity of managing immunosuppression in women of childbearing age, a common challenge in the management of Takayasu’s arteritis. It also demonstrates the importance of considering vascular complications and discusses management of these in both the acute and chronic setting. Whilst considering active inflammation and vessel damage.

• Additionally, we describe the potential options available when first line therapies are either ineffective, not tolerated or inappropriate. This case explores reproductive options to those requiring immunosuppression.

• This case also raises the ethical dilemma of advising against pregnancy and highlights the importance of a dedicated preconception service in supporting both the consultant rheumatologist and the patient through shared decision-making.

• Takayasu’s patients benefit from a MDT service to aid complex decision making. For example, rheumatology, vascular and obstetrics input.

